# Single and combined use of the platelet–lymphocyte ratio and neutrophil–lymphocyte ratio in hemorrhagic fever with renal syndrome

**DOI:** 10.3389/fcimb.2026.1744838

**Published:** 2026-05-29

**Authors:** Ting Wang, Kai Qi, Yaoni Li, Qiujian Zhao, Yazhou Yao, Lilan Zheng, Xingxing Zheng, Haodong Shi

**Affiliations:** 1 Clinical Laboratory, Baoji Central Hospital, Baoji, Shaanxi, China; 2 Clinical Laboratory, Fujian Provincial Hospital South Branch, Fuzhou, Fujian, China; 3 Radiology Department, Baoji Central Hospital, Baoji, Shaanxi, China; 4 Orthopedics Department, Baoji Central Hospital, Baoji, Shaanxi, China

**Keywords:** diagnosis, hemorrhagic fever with renal syndrome, neutrophil-lymphocyte ratio, platelet counts, platelet-lymphocyte ratio

## Abstract

**Objective:**

This study aims to evaluate the diagnostic performance of the platelet-lymphocyte ratio (PLR) and neutrophil-lymphocyte ratio (NLR) in hemorrhagic fever with renal syndrome (HFRS).

**Methods:**

This retrospective study included 215 patients with HFRS (HFRS group) and 256 healthy controls (HC group). Peripheral blood samples were collected to measure platelet counts (PLT), and PLR and NLR were subsequently calculated. Multivariate logistic regression was used to adjust for the confounding effects of age and gender. Receiver operating characteristic (ROC) curves, net reclassification improvement (NRI) and integrated discrimination improvement (IDI) assessed their predictive performance.

**Results:**

For HFRS diagnosis, the AUC of PLR (0.8141; 95% CI 0.7682-0.8600) was higher than that of NLR (0.6412; 95% CI 0.5882-0.6942). PLT count achieved the highest AUC (0.8729; 95% CI 0.8356-0.9102), specificity (94.92%) and sensitivity (76.74%). The combined PLT, PLR, and NLR achieved a significantly greater AUC (0.9029, NRI 0.234, IDI 0.002, all P < 0.05) than individual indicators. After adjusting for age and gender, PLT, PLR, and NLR remained significant independent predictors of HFRS. NLR showed a significant age - dependent increase, with older patients exhibiting the highest values.

**Conclusion:**

PLT, PLR, and NLR especially their combination, may be valuable and accessible biomarkers for the adjunctive diagnosis of HFRS.

## 1 Introduction

Hantavirus-induced hemorrhagic fever with renal syndrome (HFRS) is a severe zoonotic disease that is widely prevalent in countries across Asia and Europe. It is characterized by high morbidity and mortality, with clinical manifestations ranging from mild symptoms to acute kidney injury, coagulopathy, and hemorrhage, often progressing to multi-organ failure (Vaheri et al., 2013). HFRS is primarily transmitted through rodent excreta, with outbreaks often occurring during specific seasons due to increased rodent activity (Zou and Sun, 2020). Over the past two decades, HFRS has remained a significant public health challenge in endemic areas, with a case fatality rate reaching up to 20% in severe cases (Avšič-Županc et al., 2019). Early diagnosis is critical to manage the disease effectively and prevent severe complications, as delayed intervention is linked to worsened outcomes (Tian et al., 2020; Sehgal et al., 2023). Currently, diagnosis of HFRS relies on a combination of clinical symptoms, epidemiological history, and laboratory findings, including thrombocytopenia and renal dysfunction (Tian et al., 2020). However, the lack of specific and rapid diagnostic tests for early-stage HFRS continues to hinder prompt treatment.

With the deepening research on hematological markers in recent years, such as the platelet lymphocyte ratio (PLR) and the neutrophil lymphocyte ratio (NLR) have emerged as potential biomarkers for systemic inflammation and disease severity across various infectious and inflammatory conditions (Liu et al., 2021; Moosmann et al., 2022). Platelets (PLT) play a crucial role in the regulation of inflammation, thrombosis, and coagulation. Thrombocytopenia is a hallmark feature in many infectious diseases, including HFRS, where it correlates with disease severity and poor prognosis (Wang et al., 2013). In addition to the platelet count, the PLR has been proposed as a useful marker for inflammation. PLR reflects the inflammatory balance between platelets, which are involved in coagulation and thrombosis, and lymphocytes, which are key players in immune responses (Balta et al., 2013). Studies have shown that PLR may be an effective prognostic marker in various acute inflammatory conditions, including HFRS, as it reflects the body’s systemic inflammatory state (Zhu et al., 2023). Similarly, the NLR, which quantifies the relationship between lymphocytes and neutrophils, has emerged as another valuable marker of systemic inflammation. Elevated NLR levels have been linked to worse outcomes in several viral infections, including hantavirus-related diseases, indicating that NLR can also predict the severity and prognosis of HFRS (Fan et al., 2018; Nusshag et al., 2024). Both PLR and NLR have demonstrated significant prognostic value in inflammatory diseases, and their combined use could provide a more comprehensive diagnostic tool for HFRS (Lee et al., 2022; Yao et al., 2024).

In the study, we aimed to further clarify the relationship between HFRS and common predictors such as the PLT count and inflammation indicators such as PLR and NLR. We investigated the utility of the PLT count, PLR, and NLR, both individually and in combination, in diagnosing HFRS. This work may provide a theoretical basis and experimental foundation for the early diagnosis of HFRS.

## 2 Methods

### 2.1 Participants

The study sample was drawn from inpatients at the Baoji Central Hospital. The HFRS group consisted of patients clinically diagnosed with hemorrhagic fever with renal syndrome, while the healthy control (HC) group was composed of individuals undergoing health examinations at the same hospital during the same period. Demographic characteristics of the patients were obtained from their medical records. The exclusion criteria for this study were strictly enforced. The HFRS patients ultimately included were those with a primary diagnosis of HFRS, excluding patients with other active renal diseases, diabetes, cardiovascular diseases, hematological disorders, autoimmune diseases, viral hepatitis, and other liver diseases that could affect hematological indicators. Ultimately, 215 HFRS inpatients and 256 healthy controls were included.

### 2.2 Variables and measurements

Whole blood cell count data were obtained using the SYSMEX XN-3000 laboratory equipment (Sysmex Corporation, Japan). Quality control was completed in accordance with the operating instructions, and the experimental process met the testing requirements.

**Figure 1:**
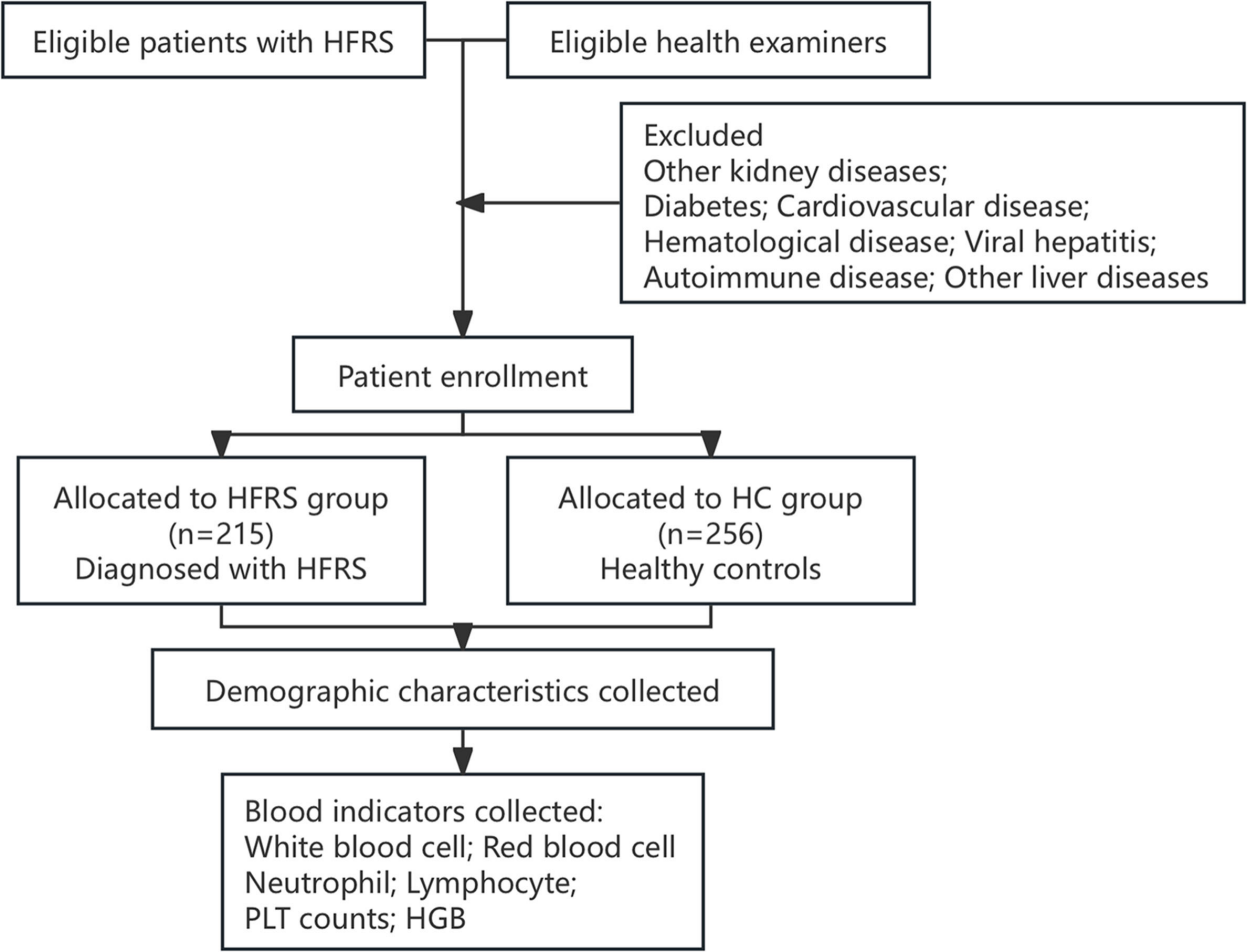
Study flow diagram.

To capture the hematological features at initial diagnosis, the blood samples analyzed in this study were the first samples collected within 24 hours of patient admission, corresponding to the acute febrile phase of the disease (typically 3–5 days after symptom onset). All blood samples were taken in the morning before breakfast, and data were collected from the laboratory’s electronic database. White blood cell (WBC), red blood cell (RBC), neutrophil, lymphocyte, and PLT counts, as well as hemoglobin (HGB) levels, were recorded, The experimental procedure is presented in **Figure 1**. PLR and NLR were calculated as follows:

PLR = PLT count/lymphocyte count.

NLR = neutrophil count/lymphocyte count.

The data to the right of the equal sign in each formula were obtained from the same test. The results were calculated to two decimal places.

### 2.3 Ethics statement

The present study was carried out in accordance with the principles of the Declaration of Helsinki. Ethical approval was obtained from the Ethics Committee of Baoji Central Hospital. Written informed consent was waived owing to the retrospective nature of the research.

### 2.4 Statistical analysis

The statistical analysis methods used in this study were conducted using SPSS version 22.0 (IBM Corporation, Armonk, NY, USA) and R software (version 4.4.1). The Shapiro-Wilk test was used to assess the normality of continuous variable data. For non-normally distributed continuous variables, median and interquartile range (IQR) were used for description, and the Mann-Whitney U test was used for comparisons between the HFRS and HC groups. The chi-square test was used for categorical variables. Predictive values were assessed using receiver operating characteristic (ROC) curves and quantified by calculating the area under the curve (AUC) and its 95% confidence interval (CI). To control for the potential confounding effects of age and gender, a multivariate binary logistic regression analysis was performed. HFRS status (case vs. control) was used as the dependent variable, while PLT, PLR, NLR, age (as a continuous variable), and gender were included as independent variables. By calculating the adjusted odds ratios (aOR) and their 95% CI, we assessed the independent diagnostic value of each marker for HFRS. To estimate the additional predictive power, the net reclassification improvement (NRI) and integrated discrimination improvement (IDI) were calculated. A two-tailed P < 0.05 was considered statistically significant.

## 3 Results

### 3.1 Laboratory indicators for both groups

**Table 1** presents the general demographic data and various blood biomarkers of the two participant groups. Compared to the HC group, the HFRS group exhibited significantly elevated WBC, neutrophil, and lymphocyte counts, as well as a higher NLR. In contrast, the PLT and RBC counts, HGB levels, and PLR were lower (all P< 0.001).

**Table 1:** Laboratory values of HFRS patients and healthy controls.

Variables	Total (n = 471)	HC group (n = 256)	HFRS group (n = 215)	*P*
AGE, years	47.08 ± 16.01	43.59 ± 14.44	51.24 ± 16.82	<0.001
Sex, n(%)				<0.001
Male	282 (59.87)	135 (52.73)	147 (68.37)	
Female	189 (40.13)	121 (47.27)	68 (31.63)	
WBC, 10^9^/L	9.01 ± 6.94	5.97 ± 1.49	12.62 ± 8.89	<0.001
PLT, 10^9^/L	166.00 (56.50, 220.00)	203.00 (171.50, 247.25)	48.00 (21.50, 110.00)	<0.001
LYM#, 10^9^/L	1.90 (1.40, 2.63)	1.80 (1.46, 2.20)	2.20 (1.38, 3.98)	<0.001
NEU#, 10^9^/L	3.90 (3.00, 5.80)	3.30 (2.86, 4.20)	5.87 (3.71, 9.12)	<0.001
RBC, 10^12^/L	4.45 ± 0.70	4.71 ± 0.49	4.14 ± 0.78	<0.001
HGB, g/L	137.06 ± 21.84	144.03 ± 16.98	128.75 ± 24.00	<0.001
PLR	90.83 (25.84, 133.00)	109.01 (89.11, 145.51)	20.00 (6.46, 79.24)	<0.001
NLR	2.05 (1.50, 2.99)	1.87 (1.44, 2.42)	2.46 (1.53, 4.36)	<0.001

t, t-test; Z, Mann-Whitney test; χ², Chi-square test.

SD, standard deviation; M, Median; Q_1_, 1st Quartile; Q_3_, 3st Quartile

### 3.2 Diagnostic value of the PLT count, PLR, and NLR in HFRS

**Figure 2:**
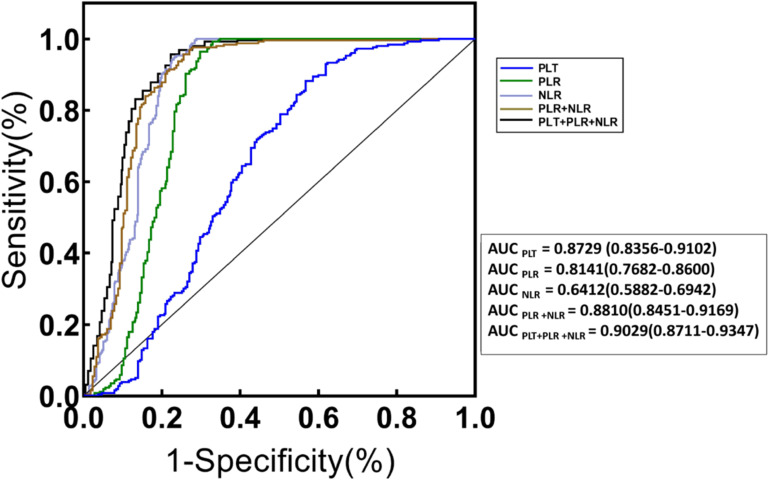
HFRS patients and healthy control were grouped to calculate the value of single and/or combined PLT, NLR and PLR for the diagnosis of HFRS. PLT, Platelet; NLR, neutrophil-lymphocyte ratio; PLR, platelet-lymphocyte ratio.

As presented in **Table 2**, the optimal cutoffs for the PLT count, PLR, and NLR were determined to be 120.5, 58.92, and 2.927, respectively, for HFRS diagnosis when the Youden index was taken as the maximum value. As presented in **Table 2** and **Figure 2**, the AUC for the PLT count (AUC = 0.8729; 95% CI = 0.8356-0.9102) was considerably higher than those of PLR (AUC = 0.8141; 95% CI = 0.7682-0.8600; P< 0.001) and NLR (AUC = 0.6412; 95% CI = 0.5882-0.6942; P< 0.001) when these variables were applied alone to diagnose HFRS. In addition, the PLT count had the highest specificity and sensitivity (94.92% and 76.74%, respectively). When PLR and NLR were combined, the AUC increased to 0.8810 (95% CI = 0.8451-0.9169; P < 0.091). When the two indicators were further combined with the PLT count, the AUC increased to 0.9029(NRI 0.234, IDI 0.002, all P < 0.05,**Supplementary Table 2**).

**Table 2:** Diagnostic efficiency of the PLT count, PLR and NLR individually and in combination for HFRS patients.

Indicator	AUC (95%CI)	Cut-off value	Sensitivity (%)	Specificity (%)
PLT	0.8729(0.8356-0.9102)	120.5	0.9492	0.7674
PLR	0.8141(0.7682-0.8600)	58.92	0.9648	0.7023
NLR	0.6412(0.5882-0.6942)	2.927	0.8828	0.4326
PLR+NLR	0.8810(0.8451-0.9169)	一	0.957	0.7442
PLT+PLR+NLR	0.9029(0.8711-0.9347)	一	0.957	0.7721

### 3.3 Independent diagnostic value after adjusting for confounders

To address the potential confounding bias from baseline differences in age and gender between the groups, we conducted a multivariate logistic regression analysis. As shown in **Table 3**, both the crude model (Model 1) and the model adjusted for age and sex (Model 2) demonstrated that PLT, PLR, and NLR were highly significant predictors for HFRS diagnosis. In the adjusted model, lower levels of PLT (aOR=0.981, 95% CI = 0.977-0.984) and PLR (aOR=0.988, 95% CI = 0.985-0.992) were independently associated with increased odds of HFRS, while higher levels of NLR (aOR=1.485, 95% CI = 1.278-1.726) were independently associated with increased odds of HFRS (all P<0.001).

**Table 3:** Multivariate logistic regression analysis for HFRS diagnosis.

Variables	Model1		Model2	
	OR (95%CI)	*P*	OR (95%CI)	*P*
PLT	0.980 (0.977 ~ 0.983)	<0.001	0.981 (0.977 ~ 0.984)	<0.001
PLR	0.987 (0.984 ~ 0.991)	<0.001	0.988 (0.985 ~ 0.992)	<0.001
NLR	1.544 (1.332 ~ 1.790)	<0.001	1.485 (1.278 ~ 1.726)	<0.001

OR, Odds Ratio; CI, Confidence Interval.

Model1: Crude.

Model2: Adjust: Sex, Age.

### 3.4 Diagnostic value of the PLT count, PLR, and NLR by patient age

**Figure 3:**
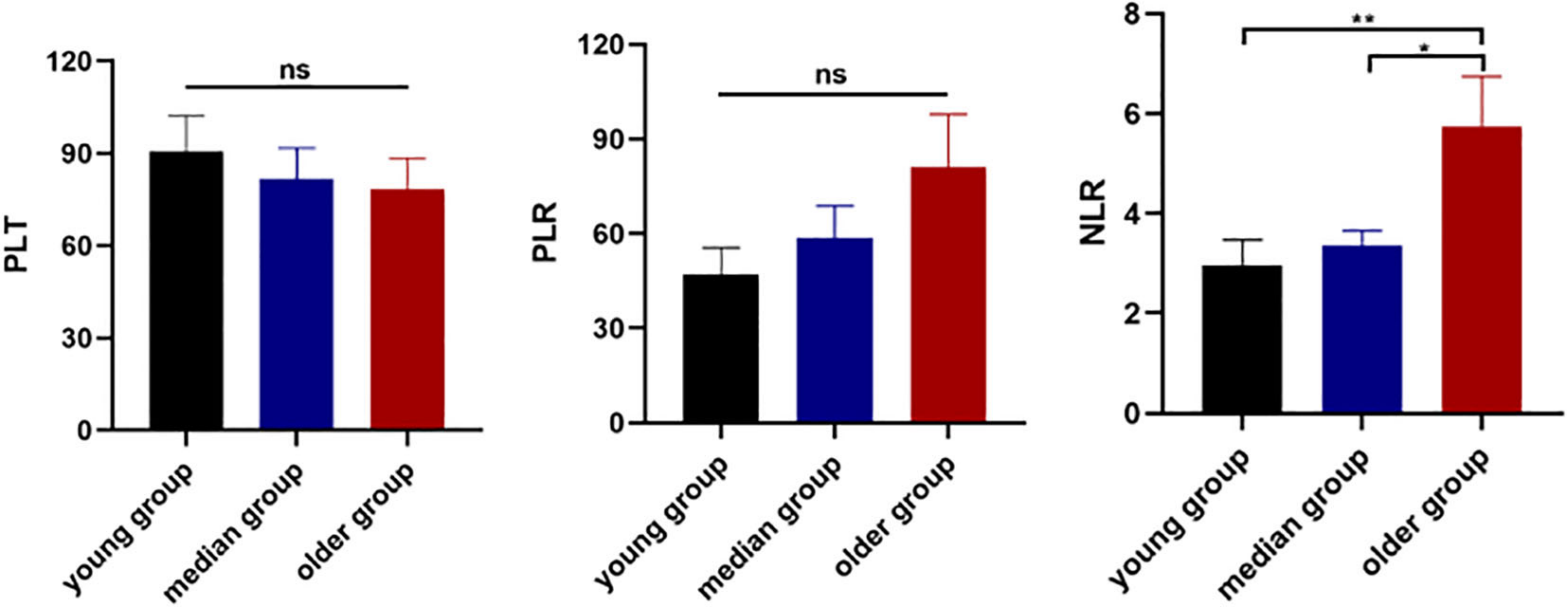
The association of the PLT **(A)**, PLR **(B)** and NLR**(C)** with the different age groups in HFRS patients. PLT, platelet; PLR, platelet-lymphocyte ratio; NLR, neutrophil-lymphocyte ratio. *P < 0.05, **P < 0.01.

To examine the effects of age on the diagnostic utility of the PLT count, PLR, and NLR, we separated patients into three groups: younger (≤40 years), middle-aged (41–59 years) and older (≥60 years). As presented in **Table 4**, more patients were classified into the older group than into the other two groups. In addition, younger patients tended to have high PLT counts. The PLT count decreased as patients aged, but the difference was not significant. There was a subtle difference in PLR among the three groups. Conversely, a significant difference in NLR was detected among the groups (P = 0.011). *Post hoc* analysis (LSD) confirmed that NLR was remarkably higher in older patients than in middle-aged (P = 0.013) and younger groups (P = 0.008). As presented in **Figure 3**, older patients tended to have high NLR, and when considering the subject status, there was a significant effect of patient age.

**Table 4:** Systemic inflammation laboratory values for different age groups.

Variable	Younger group (≤40 years)	Middle-aged group (41–59 years)	Older group (≥ 60 years)
N	58	78	79
Age (male, %)	41(70.7%)	60(76.9%)	46(58.2%)
PLT	54.00 (24.50, 141.75)	45.50 (22.25, 80.50)	48.00 (18.00, 113.00)
PLR	17.30 (5.32, 71.43)	19.63 (7.83, 64.30)	23.66 (6.13, 102.75)
NLR	2.03 (0.99, 3.02)	2.57 (1.52, 4.58)	2.93 (1.88, 5.08)

## 4 Discussion

This study aimed to investigate the diagnostic value of PLT, PLR, and NLR in HFRS, a severe infectious disease caused by hantaviruses. Our research confirms that after statistical adjustment for confounding factors like age and gender, PLT, PLR, and NLR, especially their combination, may be useful and effective auxiliary markers for HFRS diagnosis, as similarly evidenced by NRI and IDI metrics.

Consistent with previous studies, our findings validate that PLT is a critical aided diagnostic indicator for HFRS, as thrombocytopenia is a hallmark of the disease (Wang et al., 2013; Vaheri et al., 2013).The core pathogenesis of HFRS involves viral infection and damage to vascular endothelial cells (Jiang et al., 2016). This damage triggers extensive platelet activation, adhesion to the injured endothelium, and subsequent consumption, leading to a sharp decline in peripheral PLT counts (Schrottmaier et al., 2022). Although the bone marrow compensates by increasing thrombopoietin (TPO) levels and releasing immature platelets, the rapid peripheral consumption makes thrombocytopenia an inevitable early phenomenon (Connolly-Andersen et al., 2015; Laine et al., 2016). This may explains why PLT alone demonstrates high diagnostic specificity and AUC. The significant reduction in PLR further reflects the pathophysiology of HFRS. PLR integrates platelet count and lymphocyte count, and its decrease in HFRS is driven by two synergistic processes, as viral infection damages vascular endothelial cells, platelets are rapidly activated and consumed, and activated T lymphocytes extravasate from the peripheral circulation to infected tissues, resulting in a relative increase in peripheral lymphocyte count in some patients, making it a more comprehensive marker of HFRS-related vascular and immune dysfunction than single PLT or lymphocyte count alone.

In this study, we observed significantly elevated NLR in HFRS patients compared to healthy controls, consistent with recent findings in other hantavirus infection studies (Nusshag et al., 2024). Elevated NLR reflects the complex immune response during HFRS. On one hand, virus-infected endothelial cells release chemokines, such as interleukin-8 (IL-8), extensively recruiting and activating neutrophils, leading to neutrophilia (Strandin et al., 2018). While this response represents the innate immune attempt to clear the virus, it simultaneously exacerbates inflammatory tissue damage, notably within renal tissues. On the other hand, activated lymphocytes, particularly T-cells, extravasate extensively from circulation into infected tissues to effectively eliminate the virus, resulting in reduced peripheral lymphocyte counts (Resman Rus et al., 2020). Thus, the NLR, as a ratio of elevated neutrophils to reduced lymphocytes, may accurately capture this immunological imbalance driven by combined innate immune activation and adaptive immune cell migration, making it a valuable indicator of disease severity. Notably, our age-stratified data revealed that NLR increases with age. As HFRS patients in China are primarily young and middle-aged adults, accompanied by an increasing incidence in individuals aged over 60 years in recent years (Zhang et al., 2014), the enrolled patients in this analysis were stratified into three age strata: ≤ 40 years, 41~59 years, and ≥ 60 years. Older patients had a significantly higher NLR than middle-aged and younger patients. This age dependence likely stems from age-related immune. Elderly individuals have reduced neutrophil phagocytic efficiency and impaired lymphocyte proliferation, leading to a more pronounced inflammatory response imbalance during HFRS (Zhu et al., 2023; Yao et al., 2024). This mechanism explains why NLR is particularly valuable for identifying elderly HFRS patients, who are at higher risk of severe complications, by capturing their age-specific immune vulnerability.

A significant innovation of this study is the systematic evaluation of a combined diagnostic model comprising PLT, PLR, and NLR. While NLR and PLR have been employed as inflammatory biomarkers in various diseases (Eslamijouybari et al., 2020), their distinctive diagnostic value in HFRS arises from their close alignment with the disease’s core pathology. Our findings suggest that combining NLR, reflecting systemic inflammation, with two markers directly representing the hallmark platelet consumption characteristic of HFRS, results in a synergistic diagnostic model. This comprehensive model integrates three critical dimensions—platelet depletion (indicating vascular endothelial injury), neutrophil-driven inflammation (innate immune activation), and lymphocyte dynamics (adaptive immune response)—thus providing a thorough depiction of HFRS pathophysiology. Such multidimensional assessment underlies the superior diagnostic performance (AUC = 0.903) of this combined model compared to any single parameter.

During the acute infectious phase, HFRS, COVID-19 and dengue disease may exhibit considerable similarities in both their clinical presentations and laboratory findings. To our knowledge, this is the first study to verify that PLT, PLR, and NLR, especially in combination, may act as useful and effective auxiliary indicators for HFRS diagnosis. Interestingly, previous studies have indicated that in the context of overlapping outbreaks, PLT and NLR could serve as a rapid, cost-effective approach to distinguish between dengue disease and COVID-19 (Osuna-Ramos et al., 2022). Further well-designed studies are required to explore and compare the specificity and sensitivity of these peripheral blood cell count indicators for the auxiliary diagnosis of these viral infectious diseases.

The innovation of our study lies in the combined use of PLT, PLR, and NLR for diagnosing HFRS. While PLT has long been recognized as a diagnostic indicator (Wang et al., 2013; Vaheri et al., 2013), the use of PLR and NLR together with PLT offers a novel and more comprehensive diagnostic approach. This combination allows for a multi-faceted evaluation of systemic inflammation, potentially providing more accurate and early detection of HFRS, especially in settings with limited diagnostic resources. Moreover, the diagnostic utility of these markers is further enhanced when applied in different age groups, as seen with the age-related differences in NLR.

The results of this study have significant potential for clinical translation. There is currently a lack of head-to-head comparisons between serological diagnostic approaches and our diagnostic strategy. Notably, in endemic areas of this virus, diagnosis is typically initiated based on clinical manifestations and initial laboratory test results (Sehgal et al., 2023). The three indicators-PLT, PLR, and NLR-are all derived from the complete blood count (CBC), a routine, inexpensive, and rapid test available in most healthcare facilities, including resource-limited primary care units. In contrast, serological confirmation methods for HFRS, such as ELISA and immunofluorescence assay (IFA), typically require more time, higher costs, and specialized laboratory equipment. Therefore, we propose the combined evaluation of PLT, PLR, and NLR as an effective early warning and triage tool. In HFRS-endemic areas, patients presenting with symptoms like fever and fatigue who show characteristic changes in these combined indicators (i.e., decreased PLT and PLR, increased NLR) should be highly suspected of having HFRS, prompting immediate preliminary interventions while confirmatory etiological tests are being conducted. Given that NLR is age-dependently elevated and elderly HFRS patients are more likely to develop acute kidney injury or hemorrhage. For elderly patients with suspected HFRS, even if initial symptoms are mild, clinicians should prioritize: (a) close monitoring of renal function; (b) prophylactic use of anti-inflammatory agents to mitigate excessive neutrophil-mediated tissue damage; (c) avoidance of antiplatelet drugs that may exacerbate bleeding risk.

Overall, our study has certain limitations. First, the single-center and retrospective nature of this study inherently limits the external generalizability of our findings, and further multicenter prospective studies are required for validation. Due to the retrospective study design, outcome indicators such as mortality, ICU admission, and dialysis were unavailable for this cohort, limiting further exploration of the clinical utility of the proposed diagnostic criteria. This study adopted healthy individuals as the control group. Future studies incorporating patients with overlapping febrile conditions, including dengue fever, sepsis, and leptospirosis, would be more conducive to achieving valuable conclusions in diagnostic evaluation. Second, the retrospective design may introduce biases such as selection bias. We have mitigated this to some extent by using multivariate logistic regression to statistically adjust for some confounders (age and gender). However, residual confounding from key factors such as comorbidities, clinical severity, disease stage, baseline renal function, medication regimens, and concomitant infections may still affect the observed association in this study. Third, most patients included in this study were hospitalized with moderate or severe illness, as patients with mild illness would receive timely treatment in local community hospitals. The diagnostic accuracy of these markers may not be applicable to individuals in the early or mild stages of HFRS. A more complete picture of the diagnostic utility of the examined indices could be obtained if data on patients with mild HFRS can be obtained in the future. Additionally, this study categorized patients ≥60 years as a single older group. Future studies with larger sample sizes could perform a more refined subgroup analysis of this age bracket to explore whether the diagnostic value of NLR differs among older subpopulations. Fourth, owing to the absence of external validation in this study, the predictive model was particularly prone to overfitting. Finally, data on viral load, specific etiological diagnostic evidence, and immunological parameters were not incorporated in the present study. Thus, these findings are correlative and not mechanistic.

## 5 Conclusions

In summary, PLT count, PLR, and NLR may provide useful auxiliary support for the diagnosis of HFRS. The PLR, as an important inflammatory marker, may display greater utility for HFRS diagnosis when applied individually, whereas the diagnostic accuracy was improved when all three parameters were combined. NLR could be a promising index for diagnosing HFRS in older patients, who carry high risks of multiple infections and severe disease.

## Data Availability

The raw data supporting the conclusions of this article will be made available by the authors, without undue reservation.
