# Correction: Single and combined use of the platelet–lymphocyte ratio and neutrophil–lymphocyte ratio in hemorrhagic fever with renal syndrome

**DOI:** 10.3389/fcimb.2026.1918001

**Published:** 2026-07-10

**Authors:** Ting Wang, Kai Qi, Yaoni Li, Qiujian Zhao, Yazhou Yao, Lilan Zheng, Xingxing Zheng, Haodong Shi

**Affiliations:** 1 Clinical Laboratory, Baoji Central Hospital, Baoji, Shaanxi, China; 2 Clinical Laboratory, Fujian Provincial Hospital South Branch, Fuzhou, Fujian, China; 3 Radiology Department, Baoji Central Hospital, Baoji, Shaanxi, China; 4 Orthopedics Department, Baoji Central Hospital, Baoji, Shaanxi, China

**Keywords:** diagnosis, hemorrhagic fever with renal syndrome, neutrophil-lymphocyte ratio, platelet counts, platelet-lymphocyte ratio

Wrong affiliation, but not replacing with present address

Affiliation “Baoji Central Hospital” was erroneously given as “Yichang Central People’s Hospital”.

The original version of this article has been updated.

“During the language polishing process, an external editing service mistakenly added Yichang Central People’s Hospital as an affiliation. All data presented in this study were collected and analyzed at Baoji Central Hospital.”

1. A correction has been made to the section Name of Section: 2 **Methods**;

subsection Name of Sub-section: 2.1 *Participants*; Paragraph Number: 1:

“The study sample was drawn from inpatients at the Baoji Central Hospital.”

The original version of this article has been updated.

2. A correction has been made to the section Name of Section: 2 **Methods**;

subsection Name of Sub-section: 2.3 *Ethics statement*; Paragraph Number: 1:

“The present study was carried out in accordance with the principles of the Declaration of Helsinki. Ethical approval was obtained from the Ethics Committee of Baoji Central Hospital. Written informed consent was waived owing to the retrospective nature of the research. ”

The original version of this article has been updated.

