# Supplementary material for: Single and combined use of the platelet–lymphocyte ratio and neutrophil–lymphocyte ratio in hemorrhagic fever with renal syndrome
Source: Front Cell Infect Microbiol. 2026 May 29;16:1744838. doi: 10.3389/fcimb.2026.1744838 (PMC13259994; doi:10.3389/fcimb.2026.1744838)
Supplement: Supplementary file 1 [file Table1.docx]

Sample Size Calculation

This study aimed to investigate the diagnostic value of the platelet-to-lymphocyte ratio (PLR) and neutrophil-to-lymphocyte ratio (NLR) for detecting hemorrhagic fever with renal syndrome (HFRS). The sample size calculation was performed based on the expected diagnostic performance of these biomarkers using the receiver operating characteristic (ROC) curve and area under the curve (AUC) analysis.

For the calculation, we first assumed an expected AUC of 0.80, which represents a moderate effect size commonly seen in diagnostic tests. The desired power for the study was set at 80%, ensuring an 80% chance of detecting a true effect if one exists. The significance level (alpha) was set to 0.05, corresponding to a 95% confidence interval (CI) for statistical analysis.

To calculate the required sample size, the following formula for diagnostic studies was applied:

n = ((Zα/2 + Zβ) / Effect Size)²

Where:
- n = required sample size per group,
- Zα/2 = 1.96 for 95% confidence,
- Zβ = 0.84 for 80% power,
- Effect Size = expected AUC (assumed to be 0.80).

Using the formula, the minimum required sample size for each group was calculated:

 n = ((1.96 + 0.84) / 0.80)² = (2.80 / 0.80)² = (3.5)² = 12.25

Thus, the sample size per group is approximately 13 participants for each group (HFRS and healthy controls). Given the moderate effect size and the need for greater reliability in diagnostic studies, it is recommended to round up and recruit at least 20 participants per group.

The final sample size for this study includes 215 patients with HFRS and 256 healthy controls, far exceeding the minimum required sample size based on this calculation. This large sample size ensures sufficient statistical power and robustness for detecting differences in diagnostic accuracy between HFRS and healthy controls.

References
1. Stojkovic Lalosevic, M., Pavlović Marković, A., Stanković, S., et al., Combined Diagnostic Efficacy of Neutrophil-to-Lymphocyte Ratio (NLR), Platelet-to-Lymphocyte Ratio (PLR), and Mean Platelet Volume (MPV) as Biomarkers of Systemic Inflammation in the Diagnosis of Colorectal Cancer. Disease Markers 2019. DOI: 10.1155/2019/6036979
2. Fang, T., Wang, Y., Yin, X., et al., Diagnostic Sensitivity of NLR and PLR in Early Diagnosis of Gastric Cancer. Journal of Immunology Research 2020. DOI: 10.1155/2020/9146042

Table S2 Predictive performance of PLT count, combining PLT with PLR and NLR for HFRS patients.

| Indicator | AUC (95%CI) | *P* | NRI (95%CI) | *P* | IDI (95%CI) | *P* |
| --- | --- | --- | --- | --- | --- | --- |
| PLT | 0.8729(0.8356-0.9102) | Reference | Reference |  | Reference |  |
| PLT+PLR+NLR | 0.9029(0.8711-0.9347) | 0.001 | 0.234 (0.004-0.464) | 0.046 | 0.002 (0.000-0.003) | 0.047 |
